# Application of Laser-Induced Breakdown Spectroscopy for Depth Profiling of Multilayer and Graded Materials

**DOI:** 10.3390/ma16206641

**Published:** 2023-10-11

**Authors:** Agnieszka Królicka, Anna Maj, Grzegorz Łój

**Affiliations:** Department of Building Materials Technology, Faculty of Materials Science and Ceramics, AGH University of Krakow, Mickiewicza 30, 30-059 Krakow, Poland; amaj@agh.edu.pl (A.M.); gloj@agh.edu.pl (G.Ł.)

**Keywords:** laser-induced breakdown spectroscopy, multilayer material, functionally graded materials, elemental depth profiling

## Abstract

Laser-induced breakdown spectroscopy (LIBS) has emerged as a powerful analytical method for the elemental mapping and depth profiling of many materials. This review offers insight into the contemporary applications of LIBS for the depth profiling of materials whose elemental composition changes either abruptly (multilayered materials) or continuously (functionally graded or corroded materials). The spectrum of materials is discussed, spanning from laboratory-synthesized model materials to real-world products including materials for fusion reactors, photovoltaic cells, ceramic and galvanic coatings, lithium batteries, historical and archaeological artifacts, and polymeric materials. The nuances of ablation conditions and the resulting crater morphologies, which are instrumental in depth-related studies, are discussed in detail. The challenges of calibration and quantitative profiling using LIBS are also addressed. Finally, the possible directions of the evolution of LIBS applications are commented on.

## 1. Introduction

**Scheme 1:**
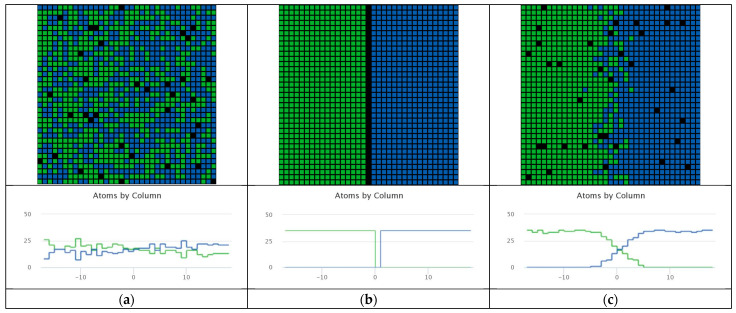
Schematic representation of materials with a different distribution of components (blue and green squares represent atoms of two different elements). (**a**) Material with an even distribution of components (composite), (**b**) material with abruptly changed composition (multilayered material), and (**c**) material with continuous transition in composition (functionally graded or corroded materials). Illustrations prepared using the simulation performed using the NetLogo Models Library [7].

Today’s advanced materials have evolved beyond simple single-component systems, embracing intricate multi-elemental compositions to achieve unprecedented properties. The pursuit of materials that can address the diverse and rigorous demands of contemporary applications has urged the development of a new generation of materials. These are not the monolithic single component materials of the past, as can be easily noticed by tracking how materials used in industries such as aviation have evolved over the years [1]. Instead, the focus has been on complex multicomponent systems that amalgamate the best attributes of their components [2]. Three categories stand out due to their innovative nature and adaptability: composites [3], multilayer materials [4], and functionally graded materials (FGMs) [5,6]. While all three categories benefit from the advantages of blending different materials, they differ in structural organization and how these materials come together (Scheme 1). The primary distinguishing feature among them is the method in which the components are merged and distributed: (i) in composites, distinct phases are combined yet remain separate, ensuring even distribution within the material (Scheme 1a); (ii) multilayered materials consist of distinct layers stacked sequentially (Scheme 1b); and (iii) in FGMs, a smooth and continuous transition in composition and/or properties is observed across the material (Scheme 1c).

**Figure 1:**
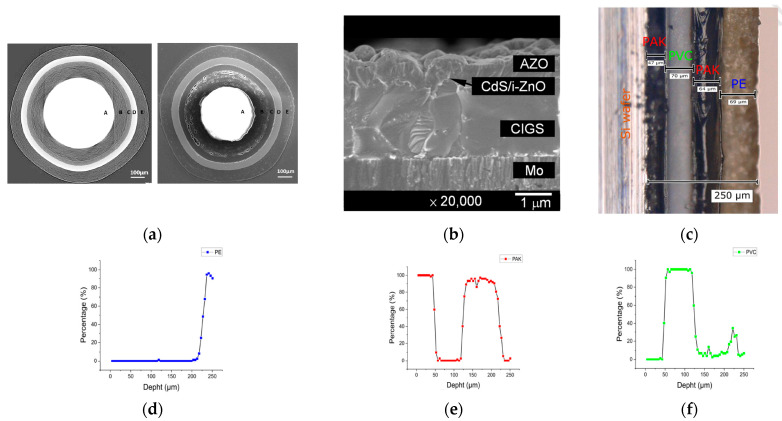
(**a**–**c**) Images of multilayer materials subjected to LIBS analysis: (**a**) spherical three-layered fuel particles used in nuclear reactors [28]. (**b**) Thin solar cell based on CuIn_1-x_Ga_x_Se_2_ (CIGS) [29]. (**c**) Double-sided adhesive tape on a Si wafer [30]. (**d**–**f**) Depth profiles obtained using LIBS for: (**d**) polyethylene, (**e**) polyacrylate, and (**f**) polyvinylchloride layers of the double-sided adhesive tape. Panels (**c**–**f**) adapted according to [30] with permission.

**Figure 2:**
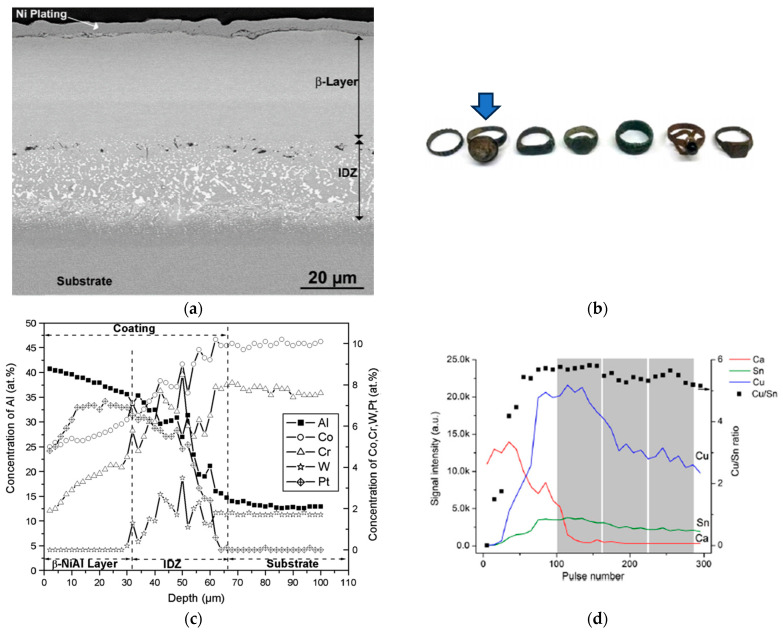
(**a**) SEM image of functionally graded material, a thermal barrier coating used in the manufacturing of jet engines (adapted according to [31] with permission). (**b**) Photograph of ancient bronze rings (adapted according to [32] with permission). (**c**,**d**) Concentration profiles obtained for functionally graded material (**c**) and ancient rings (**d**) marked with an arrow in panel b (adapted according to [31,32] with permission).

Some examples of multilayer and diffusion-controlled materials are presented in Figure 1 and Figure 2.

As the need for materials with customized properties grows, especially in high-performance applications such as nuclear reactors, electronics, and energy production and storage devices, the study and development of multilayered materials are expected to continue and expand [4]. Multilayer materials’ performance and their corrosion resistance can be influenced by the properties of individual layers, their interactions, and how they respond to environmental factors. The heterogeneity inherent in multicomponent and especially multilayered materials presents unique challenges, necessitating the development of tailored analytical techniques that can probe each layer in detail and provide a concentration profile.

Only a handful of analytical techniques can produce concentration profiles during measurements. This group includes methods in which ablation is integral to the analytical process. Ablation is commonly achieved using high-energy beams, such as those from lasers [8], ions [9], or high-energy electrons [10]. Techniques such as laser ablation inductively coupled plasma mass spectrometry (LA-ICP-MS) [11], laser-induced breakdown spectroscopy (LIBS), secondary ion mass spectrometry (SIMS) [12], and glow discharge optical emission spectrometry (GD-OES) [13] have been developed and are widely used across various laboratories. However, other methods, such as laser ionization mass spectrometry (LIMS) [14] and electron probe microanalysis (EPMA) [15] are less widespread.

**Figure 3:**
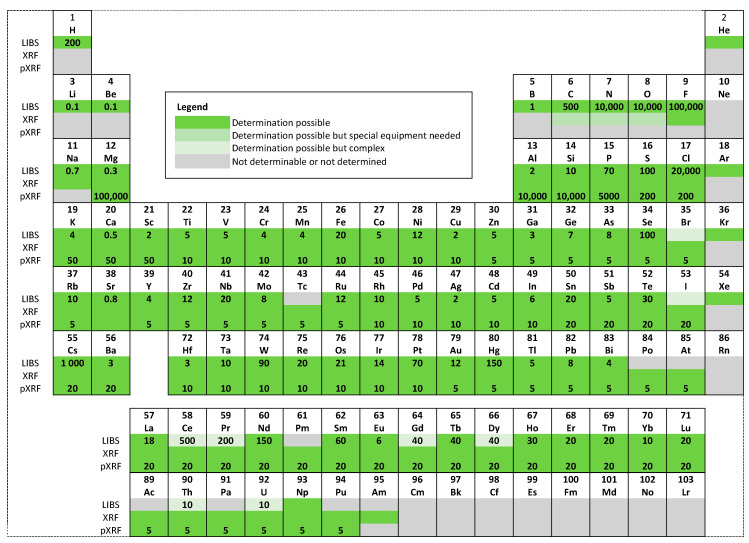
The periodic table with elements determined by LIBS, XRF, and portable XRF (pXRF) is marked in green. Limits of detection (LOD) for LIBS and pXRF, expressed in µg/g, are noted below the respective element symbols. The LODs for stationary XRF are not provided due to the varied capabilities of different XRF instruments. The LODs for LIBS, specific to individual elements, are sourced from papers [33,34] with permission. The ability of LIBS to detect Br and I is also detailed in [35]. The papers [36,37,38] present the LIBS spectra for noble gases: He, Ar, Xe, and Kr. Information on Np determination through LIBS is available in [39], while LODs for pXRF are obtained from [40] with permission.

Among the techniques mentioned earlier, LIBS stands out prominently [16,17]. LIBS is an atomic emission spectroscopy method. It utilizes a high-intensity laser pulse to ablate a minute section of the sample. By consecutively ablating the sample with multiple laser pulses and subsequently analyzing the emitted spectrum with each pulse, it is possible to gather information about elemental concentration with respect to depth. Each laser pulse strips away a thin layer, enabling the subsequent pulse to explore a slightly deeper portion. The ejected matter from this process forms a plasma plume, emitting light that is representative of the elements within the sample. LIBS can offer qualitative and quantitative assessments of the elemental composition through this emitted light analysis. Impressively, LIBS is sensitive to most elements in the periodic table. A comparative analysis of the elements detected and their respective detection limits when using the LIBS technique versus the more conventional XRF technique is represented graphically in a periodic table in Figure 3. The ablation crater’s depth can be measured using optical or electron microscopy [18], atomic force microscopy [19], or profilometry [20,21]. A depth profile of elemental concentration can be constructed by correlating the depth with the number of laser pulses and the gathered spectra.

Highlighting its adaptability, LIBS is apt for both stationary laboratory settings and on-site field applications [22]. Stationary LIBS equipment is commercially available, and there are also portable variants [23,24], analogous to widely used portable XRF analyzers. A notable recent advancement in LIBS technology is an analyzer combined with an optical microscope [25]. This integration facilitates elemental analysis while also allowing the visualization of sample details and measurement of craters created during the ablation process.

The development of the LIBS technique and its applications have been reviewed several times since 2013, when monumental work was published, covering 300+ articles [26]. Since then, several review articles have been published each year dedicated to the characterization of the LIBS technique, the development of equipment, and its applications. The published data and review articles indicate that despite the potential of LIBS for depth profiling, its predominant application remains surface mapping [27]. To emphasize the capability of LIBS to provide depth concentration profiles or stratigraphic analysis, a vital feature for multilayer materials, functionally graded materials, and diffusion-controlled processes, we have chosen to review LIBS-related articles published between 2008 and 2023. This time range allows us to trace the applications of the technique, ranging from relatively simple model samples of multilayer systems to its use in quality control performed on-site by thin-solar-cell manufacturers.

## 2. Impact of Crater Formation on LIBS Signal and Profile Analysis

The initial step in LIBS analysis involves irradiating the sample material with a high-intensity laser pulse. This action generates a microplasma above the sample and creates a distinct physical mark, an ablation crater. This crater offers valuable insights into the material’s layered composition, ablation dynamics, and the details of laser–sample interactions. When the laser beam interacts with the sample, it sharply increases the surface temperature, leading to the vaporization of a small material segment and the subsequent creation of a crater. In the context of multilayered materials, the formation of craters in LIBS serves as a “drilling” mechanism, allowing for exploring deeper layers that are typically not accessible in surface analyses. The depth directly signifies the layers accessed and profiled, which makes its geometry critical for depth-specific analyses. The structure of the crater is influenced by multiple factors, including (i) the laser parameters (like pulse width, fluence, the energy density imparted to the material’s surface during laser exposure, beam profile; focalization, etc.), (ii) the inherent properties of the material being analyzed, as certain elements can cause a sample to absorb more laser energy, leading to a deeper crater for the same laser parameters, and (iii) the surrounding atmospheric conditions (air, inert atmosphere, etc.) [8,16,17]. The composition (matrix) can influence the depth and shape of the ablation crater and, consequently, the LIBS signal. For example, certain elements can cause a system to absorb more laser energy, leading to a deeper crater for the same laser parameters.

**Figure 4:**
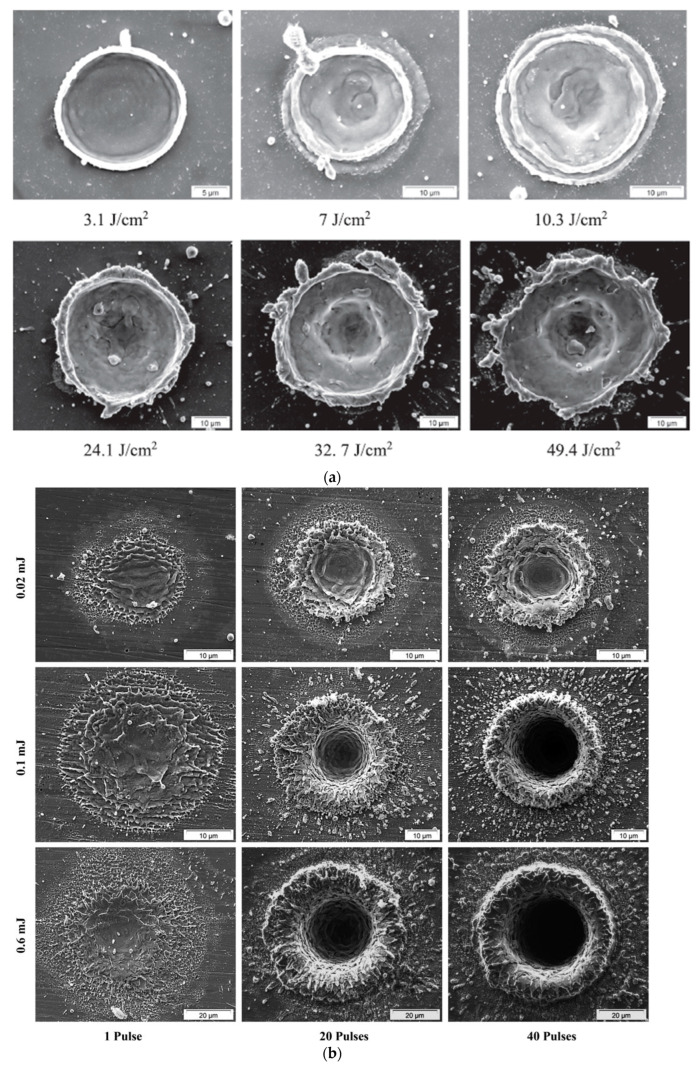

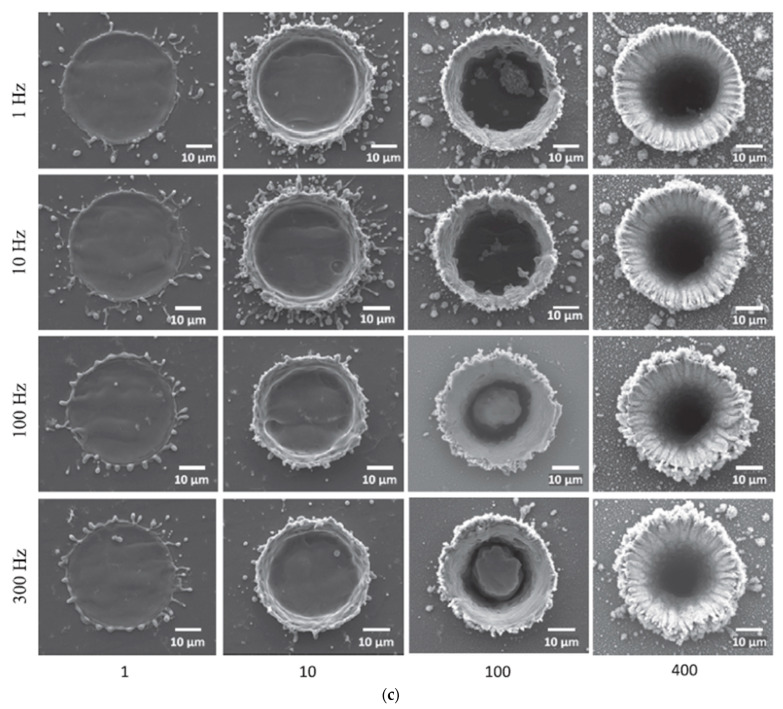
(**a**–**c**) SEM images depicting the effects of laser ablation on silicon (**a**,**c**) (adapted according to [41] with permission) and brass (**b**) (adapted according to [18] with permission) substrates under varied conditions: (**a**) variation in laser fluence, (**b**) combined variation in laser fluence and a number of laser pulses, and (**c**) combined variation in repetition rate and number of laser pulses.

In Figure 4, a gallery of craters resulting from laser ablation is presented to show the influence of various parameters on crater formation. Specifically, Figure 4a shows the effect of laser fluence, Figure 4b underscores the combined impact of laser fluence and the number of laser pulses, while Figure 4c presents the role of frequency. These illustrations are based on samples of silicone [41] and brass [18]. However, the morphology of LIBS craters is also addressed in several other studies devoted to the analysis of copper galvanized with nickel [42], galvanized steel [43], brass [44], and archeological samples of ceramics [20].

**Figure 5:**
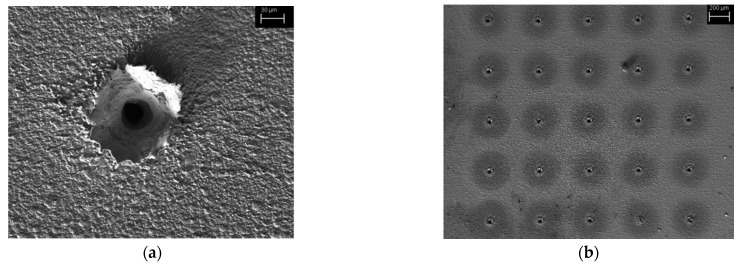

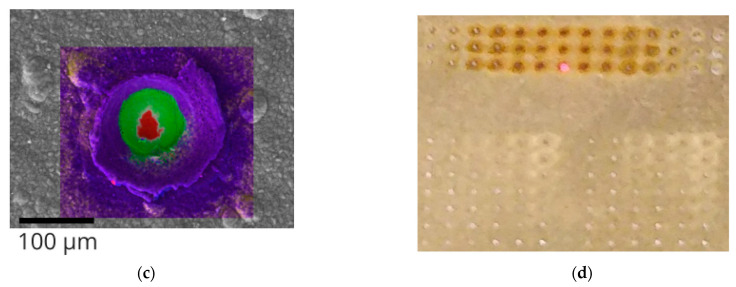
(**a**,**c**) SEM images showing a single ablation crater formed on Cu (**a**) [47] and on multilayer Al-Ni-Fe samples (**c**), where the colors indicate the abundance of the elements Al (violet), Ni (green) and Fe (red) (adapted according to [48] with permission). (**b**,**d**) Array of ablation craters formed on Cu (**b**) (adapted according to [47] with permission) and Al-Ni-Fe samples (**d**) [48].

Typically, a higher laser fluence results in a larger crater, as illustrated in Figure 4a, using silicon and brass as examples. As laser fluence increases, so does the diameter of the crater [45]. During ablation, the boiling material can cause splashes or splatters around the crater due to the expansion of the plasma, which creates a recoil pressure. These “splashes” of ablated material can distribute portions of the examined multilayered sample outside and inside the crater. This can alter the original material, affecting surface mapping (as shown in Figure 5) and depth profiling [46]. Such alterations are particularly significant when the distance between sampling points is minimal [47,48]. In the case of polymeric materials, there is an additional challenge associated with the high viscosity of the material produced by melting due to the action of the laser beam. An increase in the molecular weight of the polymer has been reported to lead to a decrease in the ablation rate of the polymer [49].

**Figure 6:**
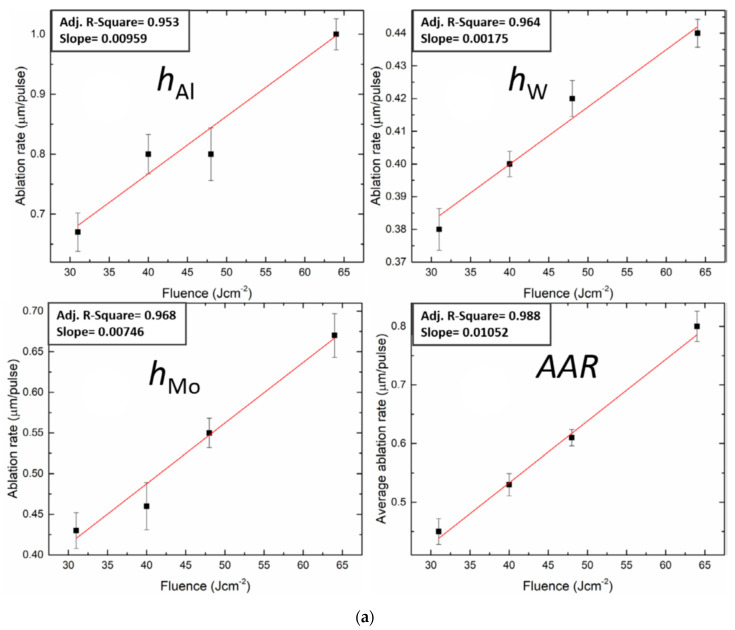

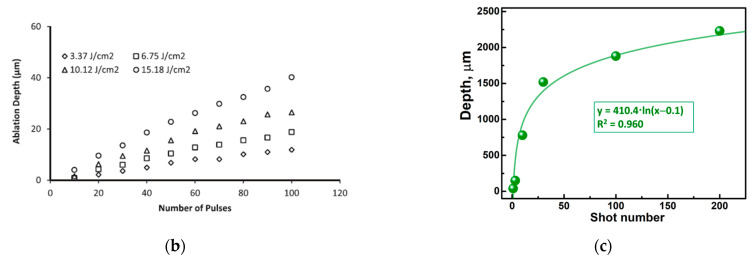
(**a**) Dependence of the ablation rates on the laser fluence for Al (h_Al_), W (h_w_), and Mo (h_Mo_); AAR corresponds to the average ablation rate (adapted according to [53] with permission). (**b**) Dependence of the depth of crater ablation of crystalline silicon on the number of laser pulses at different fluences (adapted according to [41] with permission). (**c**) Dependence of the crater depth on the number of laser pulses for the superalloy (adapted according to [52] with permission).

The typical depth resolution for LIBS depth profiling falls between 100 and 500 nm [46], although craters reaching several tens of micrometer depths [21,50,51] or even thousands of micrometer have also been documented [52]. The ablation rate is influenced by the type of material that is being ablated and the laser fluence (as shown in Figure 6a,b [41,53]), as well as by the sample temperature [21]. For consistent material and ablation conditions, the depth increases proportionally with fluence [53] and the number of laser pulses (see Figure 6b,c) [41,44]. If the material removal rate remains consistent at a given fluence, one can correlate the depth with the number of laser pulses (as depicted in Figure 6b) [41], but for deep ablation the linear range of crater depth vs. number of pulses dependance is limited as shown for aluminum [44] and superalloy samples (Figure 6c) [52]. This correlation facilitates the construction of elemental concentration depth profiles or allows for the calculation of the thickness of the material layer [54]. Although this assumption tends to be accurate for homogeneous materials and relatively shallow depths, it may oversimplify scenarios involving multilayered structures [46]. In such cases, examining the morphology and consistency of LIBS-generated craters becomes invaluable. Such studies can provide information on the varying ablation thresholds and behaviors exhibited by the different layers within a multilayered sample. Understanding and managing the dynamics of LIBS craters allows one to optimize the technique variables to provide precise depth profiles.

## 3. LIBS Spectral Lines

**Figure 7:**
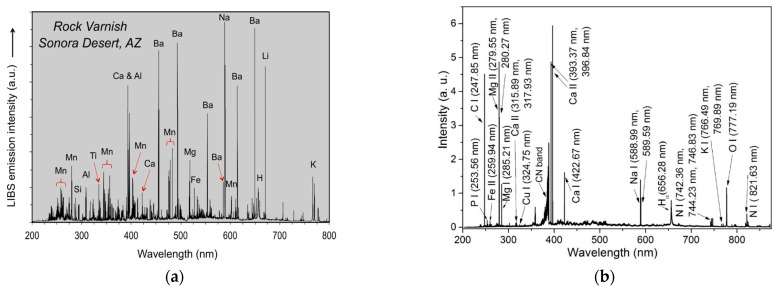

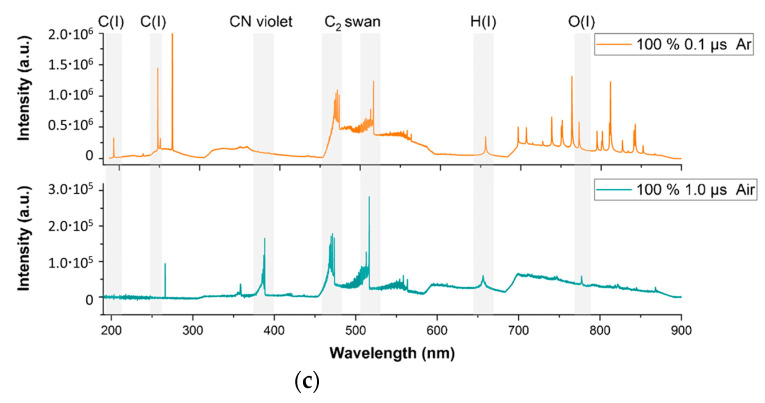
LIBS spectra obtained for (**a**) the surface of the multilayer geological sample [64], (**b**) the biological sample [65], and (**c**) the polymer sample [66].

During LIBS measurement, a laser pulse vaporizes a minute portion of the material, forming a microplasma. As this microplasma cools, the excited ions and atoms transition to their ground states. This shift from higher to lower energy levels results in the emission of photons with specific wavelengths, which are indicative of the elements in the sample. These emitted photons, ranging in wavelengths from ultraviolet to microwave, are subsequently collected and spectrally dispersed by a spectrometer. Figure 7 shows exemplary spectra across these wavelengths. The distinct spectral “fingerprint” rendered by LIBS allows for both qualitative and, with proper calibration, quantitative identification of elements in the analyzed material. It should be noted that, as shown in Figure 7b,c, LIBS reveals not only atomic bands, but also molecular bands [55,56], such as those produced by CN (cyanogen) and C_2_ (swan bands), OH [57] or AlO [58]. In the LIBS process, the characteristic emission bands of CN become apparent when carbon reacts with nitrogen in the generated plasma. These bands serve as a valuable tool for detecting materials containing carbon. If the CN band fades after the initial LIBS laser pulse, it suggests that the sample was coated with a layer containing carbon compounds, as described in the antique bronze sculpture [59]. Such observations can act as indicators of surface contamination or be used to evaluate the effectiveness of surface cleaning procedures [59,60]. In particular, these molecular bands, including C_2_ and CN, are frequently used in polymer studies [61,62,63].

## 4. LIBS Application for Studies of Multilayered Materials, Functionally Graded Materials, and Materials Affected by Diffusion-Driven Degradation Processes

Table 1 provides an overview of the multicomponent samples analyzed using LIBS. These samples span a wide range of materials tailored for extreme environments, such as those found in nuclear reactors [53,67,68,69,70,71] or materials that come into contact with plasma [54], molten salts [37,40], and solutions that arise during the purification of fissile materials [72]. They also include materials that withstand severe thermal and mechanical loads [31,73,74]. Another set featured in the table comprises superalloy composites [75], metallic coatings [32,43,47], silicon-based photovoltaic cells [45], thin film varieties of solar cells [76,77,78], lithium electrodes [79], and thin catalyst layers deposited on various substrates [80,81]. Furthermore, the table also presents historically significant materials, including archaeological artifacts [20,59,82,83], artwork [59], structural elements from heritage sites [84,85,86], and formulations used to preserve ancient masonry [87].

The study of functionally graded materials further highlights the effectiveness of LIBS in examining elemental concentration profiles. With LIBS, it was feasible to identify the presence of single-component layers and, more critically, determine the extent of a transitional layer with a composition influenced by diffusion. Figure 2c offers a vivid illustration of LIBS’s capabilities in analyzing systems with varied compositions. Furthermore, LIBS proves to be instrumental in studying corrosion processes. As illustrated in Figure 2d, LIBS enables not only the identification of the composition of the corroded layer but also an assessment of its reach.

The application of LIBS for depth profiling in polymeric materials is rare (see Table 2). However, this trend may shift soon. As suggested by a study from 2020 [30], LIBS can effectively investigate multilayer polymeric materials, such as that shown in Figure 1c. The concentration profiles obtained using LIBS for multilayer materials depicted in Figure 1d–f, coupled with microscopic images of the sample analyzed, confirm that the LIBS technique is highly effective in the study of multilayer materials, as all individual layers of various compositions were precisely identified [15]. However, achieving accurate depth profiling requires the appropriate calibration and optimization of the measurement parameters.

## 5. Analytical Performance of LIBS for Elemental Profiling

When constructing elemental depth profiles of multilayered materials, the dependence of the intensity of the spectral line of interest is typically plotted against the number of laser pulses (as shown in Table 1). Concentration profiles were reported in fewer than 20% of the experiments described in Table 1 and Table 2. For calibration, classic calibration curve methods were predominantly used [75,76,77,80], although the calibration-free approach (CF-LIBS) was also applied in its traditional [81] and modified versions (one-line calibration-free LIBS, OLCF-LIBS) [89]. Despite its undeniable benefits, the internal standard method was used in a single study [79]. Multivariate statistical methods, such as principal component analysis (PCA) and clustering of K-means, have proven to be highly effective in determining the content of individual components in multilayered materials [30]. In the following, a brief overview of the challenges and considerations associated with quantitative analysis using LIBS is presented. These include aspects related to calibration, matrix effects, instrumental parameters, and the complexities of interpreting LIBS spectra.

### 5.1. Classic and Calibration-Free LIBS

As indicated by the studies presented in the aforementioned works, quantitative elemental analysis performed using LIBS is a challenging task because of the multiple factors that influence the intensity of a LIBS signal. These range from laser-specific parameters like pulse energy, duration, wavelength, and the distance between the laser and the sample to properties of the sample’s surface, such as contamination levels and surface roughness. Additionally, the composition of the sample matrix can also introduce spectral interference, making calibration quite complicated.

However, the right calibration approach can mitigate these challenges and lead to accurate results. Traditional calibration strategies, such as the construction of calibration curves based on external standards, are often employed. Many elements exhibit numerous LIBS emission lines with distinct intensities. As a result, these lines can produce calibration curves with varied slopes. For instance, several linear regression calibration curves for phosphorus [100], chromium [101], and cerium [102] have been documented. Beyond single-variable calibration, multivariable regression models can be used that incorporate multiple signals from the LIBS spectrum. Different calibration variants, some incorporating chemometric tools, are discussed in reviews such as [102]. When choosing spectral lines for calibration, it is crucial to ensure that the chosen line is free from interference from coexisting elements. For example, although the Al I line at 309.27 nm shows a higher intensity, it is not preferred for aluminum determination due to the potential interferences of Mg I lines at 309.10, 309.29, and 309.68 nm [100]. Spectrum processing methods, such as smoothing, can enhance the shape of the calibration curve [101].

However, the use of calibration curves, even those constructed with reference materials that have a matrix composition similar to that of the sample, may not always be sufficient, especially for specific materials, such as geological samples [101]. In such situations, the incorporation of an internal standard method is beneficial. When experimental parameters change, the ratio of spectral line intensities is applied for calibration. This approach was presented for analysis of a thin solar cell film, where the In/Cu, Ga/In, and Se/In intensity ratios were used for calibration and finally concentration depth profiling [76].

In scenarios where traditional calibration is unfeasible, such as in remote-sensing applications in space missions, or when suitable standards are inaccessible or difficult to formulate (as in the analysis of unknown or complex materials), employing calibration-free LIBS (CF-LIBS) seems to be a promising solution. In CF-LIBS, the analyte concentration is calculated directly from the LIBS spectrum by relying on the physics of the laser-induced plasma. The method involves calculating the plasma temperature and electron density directly from the spectrum. These parameters, combined with atomic data, facilitate the determination of the analyte’s concentration. However, the successful application of CF-LIBS necessitates the fulfillment of specific criteria: (i) congruent mass transfer from the solid to the plasma, (ii) local thermodynamic equilibrium within the plasma, (iii) spatially uniform plasma temperature and density distributions, and (iv) negligible self-absorption of spectral lines. If these conditions are met, then the measured line intensity is proportional to the emission coefficient [103]. Utilizing an array of spectral lines corresponding to varied energy levels enables calculation of each sample element’s concentration without conventional calibration. Achieving accurate relative concentrations with CF-LIBS is difficult due to various influencing factors, such as matrix effects, laser parameters, and experimental configurations. To mitigate these problems, several adaptations of CF-LIBS have been proposed [103]. Recent review publications [104,105] provide a detailed discussion of foundational concepts, their influencing factors, analytical efficacy, and potential measurement uncertainties.

An interesting variant of CF-LIBS worth mentioning is the one-point calibration method. Using only one calibration point, it introduces an empirical correction to the purely theoretical CF-LIBS approach, helping to determine essential experimental and spectroscopic parameters, which are normally not easy to obtain [104].

### 5.2. Limits of LIBS Detection of LIBS

The limit of detection (LOD) in elemental analysis is the minimum elemental fraction for which the analytical signal can be distinguished from the background signal within a stated probability. Assuming a normal distribution of the background signal fluctuation, the limit of detection within a confidence interval of 99.7% is given by Equation (1) [106].

LOD = 3σ/S (1)

where σ is the standard deviation of the background signal and S is the sensitivity given by the slope of the calibration curve.

The calculated limits of detection (LOD) for most elements are shown in Figure 3. Although determining the LOD may seem straightforward at first glance, in the context of LIBS it is a complex task [107]. The complexities arise from various factors, including the calibration methods (univariate, multivariate) [107] and the problems specific to the calibration-free LIBS approach [104,108].

## 6. Conclusions

Laser-induced breakdown spectroscopy has emerged as a promising technique for the rapid, non-destructive, or micro-destructive depth profiling of multilayer materials with well-defined layers and diffusion-driven materials. As this review has shown, with its ability to produce elemental maps and profiles, LIBS offers an unparalleled tool for studying the surface and deeper layers of multilayer materials. When LIBS results are confronted with traditional microscopic techniques, there is clear evidence of the reliability of the technique. LIBS has showcased a broad applicability for multilayer organic and inorganic materials, functionally graded materials, and materials affected diffusion-controlled processes such as corrosion. However, like any analytical tool, the effectiveness of LIBS hinges on the optimal calibration and fine-tuning of the measurement parameters.

## 7. Future Directions in LIBS Applications

As technology evolves, it can be anticipated that more sophisticated LIBS equipment will be introduced that may reduce the need for extensive multipoint calibration by employing calibration free approach, offer a higher spatial resolution, or introduce faster data acquisition speeds. Analyzing the data presented in Table 1, it can be expected that the applications of LIBS in multilayered materials research will evolve following the directions indicated below.

1. Remote measurements. In LIBS measurements, the analytical information comes from radiation emitted by elements in plasma, not from particles reaching the detector, as is the case, for example, with LA-ICP-MS. This feature enables remote analysis, a feature currently utilized in the study of the Martian surface. Remote analysis is invaluable for investigating materials that operate under extreme conditions or for situations where direct contact with the material would expose the operator to harmful radiation. Future applications are expected to include the use of LIBS to monitor the progress of wear and corrosion in various types of materials used for the construction of nuclear reactors.
2. Industrial at-line applications. LIBS measurements do not require vacuum chambers or direct contact with the sample, provide data on elemental composition quickly, and are easily automated. This makes the technique a promising tool for quality control in industries that use multilayered materials in the production of displays, monitors, thin-film photovoltaic cells, coatings, and graded wear- and heat-resistant materials.
3. Identification of materials of unknown composition. The use of calibration-free LIBS allows the identification and quantification of elements in materials with unknown compositions. Waste management, particularly recycling processes, is an anticipated application area for LIBS. This is especially crucial for multilayer materials, where valuable or toxic constituents might be embedded, making them inaccessible for surface-only analyses. By creating a depth profile via the ablation of micro-areas within the waste, LIBS facilitates the confirmation or elimination of undesirable components. This eliminates the need for costly and energy-consuming sample fragmentation, a particularly challenging process with polymeric or composite materials. Such materials include laminates, substances layered with organic and inorganic coatings, electronic waste, used photovoltaic panels, and even microplastics [109].
4. Development of integrated analytical platforms. Hybrid systems where LIBS is integrated with other analytical tools (e.g., Raman-LIBS systems, microscopes coupled with LIBS as analogous to SEM-EDS systems) could be developed to provide complementary data, enhancing the analytical capability of single-mode devices.
5. Development of laser cleaning devices equipped with a LIBS analyzer. LIBS analyzers can be integrated with laser surface cleaning devices, as laser radiation used to remove the outer layers of material can also serve as the radiation inducing the LIBS effect. This enables real-time monitoring of the efficiency of removing layers with varied chemical compositions by tracking changes in an indicator element specific to the ablated material. This capability can be utilized to track the removal of external layers during the process of ablation of worn protective coatings in aviation and seems to be usable in the cleaning of historical artifacts.

## Figures and Tables

**Table 1:** List of applications of the LIBS technique in the studies of inorganic multilayer materials, functionally graded materials, and corrosion studies employing commercial LIBS instruments (both stationary and portable) and laboratory-made LIBS systems.

No	Purpose of Studies	Analyte ^1^	Sample	Mode	Ref. Analytical Methods	Type of LIBS Device	Ref.
1	Measurement of the thickness of multilayer metallic coatings of wall materials used in plasma reactors for nuclear fusion	Al, Fe, Mo, W	Al-W-Mo layer coated on steel	Depth profiling (normalized intensity vs. number of pulses)	-	Commercial LIBS system	[53]
2	Examination of samples simulating internal walls of the fusion reactor, contaminated with traces of nuclear fuel	Al, C, D (deuter), Mo, Ti, W,	C and W-Al-C layers coated on W, Mo or Ti (Al used instead of toxic Be)	Mapping and depth profiling (intensity vs. number of laser pulses)	-	Laboratory-made LIBS system	[67]
3	Examination of graphite tiles with a marker layer of C and a deposited Mo interlayer (C-Mo-C) removed from a fusion reactor for post-operational analysis	C, H, Mo	Graphite tiles	Depth profiling (intensity vs. number of laser pulses)	LIA-QMS	Laboratory-made LIBS system	[68]
4	Examination of graphite tiles with a marker layer of C and a deposited Mo interlayer (C-Mo-C) removed from a fusion reactor for post-operational analysis	C, H, Mo, Na	Graphite tiles	Depth profiling (normalized intensity vs. number of laser pulses)	-	Laboratory-made LIBS system	[69]
5	Examination of doped graphite tiles removed from a fusion reactor for post-operational analysis	C, Ca, Cr, Fe, K, Li, Mo, Na, Si, Ti, W	Doped graphite (B_4_C, Si, Ti) tiles with SiC coating	Mapping and depth profiling (intensity vs. number of laser pulses)	EDX	Modified commercial LIBS system	[70]
6	Examination of graphite tiles removed from a fusion reactor for post-operational analysis	Cr, Fe, Ni,	Unexposed and used graphite plates	Depth profiling (intensity vs. number of laser pulses)	-	Laboratory-made LIBS system	[71]
7	Examination of graphite tiles removed from a fusion reactor for post-operational analysis	Al, C, Ca, Cr, Cu, Fe, Mn, Ni, Si	Graphite tiles	Depth profiling (correlation coefficient vs. number of laser pulses/depth)	-	Laboratory-made LIBS system	[88]
8	Examination of applicability of LIBS for the determination of the erosion/deposition behavior of the plasma-facing materials used in fusion reactors	Cu, Ni	Laboratory-made Ni–Cu multilayer plates	Depth profiling (normalized intensity vs. number of laser pulses; concentration vs. number of laser pulses; correlation coefficient vs. number of laser pulses)	-	Laboratory-made LIBS system	[89]
9	Examination of graphite tile exposed to He and H plasma in the initial operational phase of the stellarator of a nuclear fusion reactor	C, H, Na, Fe, O	Graphite tiles before and after plasma exposure	Depth profiling (intensity vs. number of laser pulses)	EDX	Laboratory-made LIBS system	[90]
10	Examination of samples simulating graphite elements of a molten salt reactor for nuclear fission exposed to a molten salt mixture consisting of lithium fluoride, sodium fluoride, and potassium fluoride (FLiNaK) or a mixture of lithium fluoride and beryllium fluoride (FLiBe)	C, F, H, He, K, Li, Na, O, (C_2_ Swan)	Graphite parallelepipeds exposed to molten Salt mixtures FLiNaK or FLiBe; LIBS measurements performed in He atmosphere	Mapping and depth profiling (relative intensity vs. number of laser pulses)	-	Commercial LIBS system	[36]
11	Examination of samples simulating steel walls of molten salt reactor for nuclear fission exposed to liquid lithium	Ar, C, Cr, Fe, Li, Mn, Ni	Welded stainless steel plates exposed to liquid lithium; experiments performed in an Ar atmosphere	Depth profiling (normalized intensity vs. number of laser pulses)	-	Laboratory-made LIBS system	[37]
12	Examination of samples simulating spherical three-layered fuel particles (TRISO) used in nuclear reactors with ZnO_2_ as a surrogate to uranium	C, O, Y, Zr	Spherical kernel made from yttria stabilized zirconia (525 um diameter) covered with pyrolytic carbon layer and outer ZrC layer	Mapping and depth profiling (normalized intensity vs. number of laser pulses)	EDS	Laboratory-made LIBS system	[91]
13	Examination of steel exposed to solutions simulating liquid solutions resulting from the plutonium uranium extraction (PUREX) process and storage pond waters	Cr, Cs, O, Sr	Stainless steel plates exposed to Cs, Sr solution in nitric acid or NaOH	Mapping and depth profiling (normalized intensity vs. number of laser pulses)	GD-OES, EDX	Laboratory-made LIBS system	[72]
14	Examination of laboratory-made laser cladded corrosion resistance hard coatings on steel support	C, Cr, Fe, Ni, W	Low alloy steel plates covered with an interlayer of nickel-based superalloy and an outer layer comprising tungsten carbide in a nickel alloy matrix	depth profiling (intensity vs. number of laser pulses/depth)	EDX	Laboratory-made LIBS system	[73]
15	Examination of laboratory-made laser cladded protective coatings with a high abrasive wear resistance on a steel support	Cr, Fe, Ni, W	Stainless-steel plates covered with NiCrBSi alloy-WC (tungsten carbide) composite	Mapping, depth profiling (normalized intensity vs. distance)	-	Laboratory-made LIBS system	[74]
16	Examination of a laboratory-made multilayered thermal protective barrier deposited on a superalloy support	Al, Co, Cr, Ni, Ta, W	Ni-based superalloy plate covered with an interlayer of nickel-aluminum alloy (β-(Ni,Pt)Al) and an outer layer comprising zirconia stabilized by yttria	Depth profiling (normalized intensity vs. depth)	EPMA	Laboratory-made LIBS system	[31]
17	Examination of laboratory-made coatings for plasma-facing components in nuclear fusion reactors	C, Cr, Cu, W	CuCrZr alloy plate coated with W	Depth profiling (intensity vs. number of laser pulses or intensity ratio vs. number of laser pulses)	EDX	Laboratory-made LIBS system	[54]
18	Examination of a laboratory-made Cu-Sn alloy similar in composition to that used in ancient Greece exposed to corrosive environments to obtain patina on bronze	Cu, Sn	Cu-Sn alloy exposed to corrosive conditions simulating acid rain, SO_2_ rich atmosphere, wet and dry cycles	Depth profiling (intensity ratio vs. number of laser pulses)	EDX, Raman	Laboratory-made LIBS system	[92]
19	Examination of laboratory-made superalloy composite powders prepared to investigate the diffusion and migration of elements in a composite powder during mechanical mixing (ball milling)	Al, Co, Cr, Mo, Ti, W	Composite metal powder pressed into tablets	Mapping, depth profiling (concentration vs. number of laser pulses)	XRF, ICP-OES	Commercial LIBS system	[75]
20	Examination of commercial samples of pure metals (Fe, Zn) and galvanized steel, laboratory-galvanized steel used for evaluation of the LIBS technique	Fe, Zn	Pure Zn, Fe plates, commercially and laboratory galvanized steel plates with variable Zn thicknesses	Depth profiling (peak area ratio vs. number of laser pulses)	-	Laboratory-made LIBS system	[43]
21	Examination of commercial samples of Ni, Cu coated steel were used to test applicability of using LIBS as a fast and well controllable tool for layer thickness analysis	Cu, Fe, Ni	Commercial steel sheets with electroplated Ni and Cu	Depth profiling (correlation coefficient vs. laser pulse number)	EDX	Laboratory-made LIBS system	[47]
22	Examination of the suitability of the hybrid LIBS-Raman system for testing archeological (bronze rings) and industrial samples representing single and multilayers materials, both inorganic and organic	Ag, Ca, Co, Cr, Cu, Fe, Mo, Ni, Pb, Sn, W, Zn	High temperature alloy, stainless steel, high temperature alloy covered with stainless steel; PE, PTFE, and titanium dioxide nanoparticles coated metal; archeological bronze rings	Depth profiling (normalized intensity vs. number of laser pulses)	reference materials NIST 1242 (high-temperature alloy) and NIST 129 (stainless steel)	Laboratory-made hybrid LIBS–Raman system	[32]
23	Characterization of laboratory-made nano-scaled (10–250 nm) layered materials applied in digital display and photovoltaic industries	Ag, Al, Ca, In, Si, Zn, (CN)	ITO layer deposited on Si_3_N_4_ using a OCP/PET as a support; PEDOT:PSS deposited on layers of Al_2_O_3_ and ZnO separated by a layer of Ag; ITO layer deposited on a glass substrate	Depth profiling (normalized intensity vs. number of laser pulses)	-	Laboratory-made LIBS system	[93]
24	Examination of commercial multicrystalline silicon (mc-Si) and laboratory-made collar cells using mc-Si were examined by LIBS to assess its applicability in quality control of mc-Si wafers and solar cells based on mc-Si	Ag, Al, Ca, H, K, Mg, Na, Si	mc-Si wafers, complete solar cell comprising front n+ junction (P-doped), antireflective/passivation layer of silicon nitride (SiNx:H), front (Ag) and rear (Al) contacts	Depth profiling (intensity vs. number of laser pulses)	-	Commercial system and laboratory-made LIBS system	[45]
25	Examination of commercial thin solar cells based on CuIn_1-x_Ga_x_Se_2_ (CIGS) tested to assess the applicability of LIBS to control (Ga + In)/Cu and (Cu + In + Ga)/Se ratios in CIGS at manufacturing sites	C, Cr, Fe, Li, Mn, Ni	Commercial CIGS solar cells with a transparent conductive layer and buffer layer that were removed by dipping in a dilute hydrochloric acid	Mapping, depth profiling (intensity vs. number of laser pulses; concentration vs. number of laser pulses)	ICP OES, AAS	Commercial LIBS system	[77]
26	Examination of commercial CIGS solar cells and a laboratory-deposited thin film of CIGS tested to assess the applicability of LIBS for elemental composition of CIGS cells	Cu, Ga, In, Mo, Se	Semiconductor Cu(In,Ga)Se_2_ thin layer deposited on Mo-coated soda-lime glass substrates; commercial CIGS solar cells with a transparent conductive layer and buffer layer that were removed by dipping in a dilute hydrochloric acid	Mapping, depth profiling (concentration vs. depth)	AES, SIMS, ICP OES, AAS	Commercial LIBS system	[76]
27	Examination of laboratory-made multilayered organic solar cells comprising a top electrode (Al, Mg or Mo), organic layer, bottom electrode (indium tin oxide), silicon nitride barrier layer, and substrate layer (polymer PET) were prepared to assess LIBS ability to distinguish the individual layers of photovoltaic coatings	Al, In, Mo, Si, Sn, (CN)	Complete 6-layered solar cells samples and samples after the removal of 1–3 layers	Depth profiling (intensity vs. number of laser pulses)		Laboratory-made LIBS system	[78]
28	Examination of laboratory-made nickel manganese cobalt (NMC) lithium-ion electrodes used to study chemical degradation mechanisms and the impact of 3D electrode architectures on cell lifetime	Li	Cathodes in the form of sheets were made in a four-stage process using Li(NiMnCo)O_2_, PVDF and conductive carbon black	Mapping, depth-profiling (concentration vs. depth)	ICP-OES	Laboratory commercial LIBS analyzer	[79]
29	Examination of laboratory-made porous Al_2_O_3_ materials manufactured to simulate the supports of hydrodemetallization catalysts used in refining petroleum to remove metal contaminants	Al, C, Cu	Cylindrical asphaltene-impregnated alumina supports	Depth profiling (concentration vs. distance)	-	Laboratory-made LIBS system	[80]
30	Examination of laboratory-prepared alumina thin films deposited on silicon wafers exhibiting catalytic properties (γ phase)	Al, Si, (AlO)	Al_2_O_3_ amorphous and crystalline thin films deposited on silicon wafers	Depth-profiling (intensity vs. number of laser pulses; concentration vs. number of laser pulses)	-	Commercial LIBS analyzer	[81]
31	Examination of laboratory-prepared mixtures of gypsum, anhydrite, and acetylsalicylic acid for the evaluation of the usefulness of the hybrid Raman/LIBS spectrometer employing double pulse arrangement (first, low-energy pulse used for Raman; second, high-energy pulse for LIBS)	Ca, Na, S, (C_2_ Swan), (CN)	Acetylsalicylic acid (Raman) and copper (LIBS) analysis in a gypsum matrix	Depth-profiling (intensity vs. number of laser pulses)	-	Laboratory-made hybrid LIBS/Raman system	[94]
32	Examination of the influence of the change in laser-to-sample distance on the performance of LIBS systems using model and archeological samples	Au, Cu, Hg	Gold and silver thin films deposited onto copper substrates; gold amalgam coated archeological decorative copper object	Depth-profiling (normalized intensity vs. number of laser pulses)	XRD	Laboratory-made LIBS system	[82]
33	Examination of the capability of LIBS systems for historically significant materials	Ag, Al, Au, Ca, CN, Cu, Hg, Mg, Na, Si, Ti	Samples taken from historic religious sites—fresco, terra-cotta, gilded bronze figure	Mapping, depth-profiling (intensity vs. number of laser pulses)		Laboratory-made hybrid LIBS–Raman system	[59]
34	Laboratory-prepared model multilayers samples representing various painting techniques using pigments of known composition to test the usefulness of LIBS in the analysis of easel paintings	Ca, Cu, Fe, Mg, Sr	Painting were prepared applying mixtures of powdered pigments and organic binders on a wooden support	Depth-profiling (intensity vs. depth)	XRF	Commercial LIBS system	[50]
35	Examination of the applicability of LIBS in the monitoring of the removal of multilayer paint coatings used in aircraft industry by laser cleaning	Ba, Cr, Sr	Carbon-fiber-reinforced plastic support covered with 4 layers of commercial paints used to protect the exterior of aircraft (polyester filler, antistatic coating, primer, topcoat)	Depth profiling (intensity vs. depth)	ICP-OES	Laboratory-made LIBS system	[95]
36	Examination of corroded archaeological copper-based artefacts to assess the applicability of LIBS in conservation and restoration of metallic objects	Ag, Au, Ba Sr, C, Ca, Cr, Cu, Fe, Mg, Mn, Na, Pb, Si, Sn, Ti, Zn	Corroded copper threads, coins, and decorative objects	Depth-profiling (normalized intensity vs. number of laser pulses), monitoring of cleaning	-	Laboratory-made LIBS system	[96]
37	Examination of the applicability of LIBS for of archaeological pottery artefacts	Al, Ca, Fe, K, Li, Mg, Na, Si, Ti	Archaeological ceramic artefacts	Mapping, depth profiling (intensity ratio vs. number of laser pulses)	XRF	Commercial laboratory LIBS system	[20]
38	Examination of archeological ceramic artefacts to characterize ancient pigments and distinguish the origin of the clay used for pottery	Al, Ba, C, Ca, Cr, Cu, Fe, K, Li, Mg, Mn, Na, Ni, Rb, Si, Sr, Ti, Zn, V	Pottery shards (objects of artistic or historic interest)	Depth profiling (intensity vs. number of laser pulses)	ICP-OES	Laboratory-made LIBS system	[83]
39	Examination of the chemical composition of the mirror matrix to obtain a degradation profile and propose hypotheses explaining the decay, which are useful for conservation efforts	Al, Ca, K Mg, Na, Si	Archaeological objects—wall mirrors—tested on-site in Pompei	Mapping, depth-profiling (intensity vs. number of laser pulses)	pED-XRF	Portable LIBS analyzer	[86]
40	Examination of mosaics to identify the materials used and assess the extent of the degradation processes caused by exposure to rainwater and the gases present in polluted air	Ca, Fe	Archaeological artefacts—mosaic tesserae—tested on-site in Pompei (Italy)	Depth-profiling (intensity vs. number of laser pulses)	pED-XRF	Portable LIBS analyzer	[85]
41	Examination of ancient Buddhist art murals for the identification of pigments used and measurement of the thickness of paint layers	Al, Ca, Cu, Fe, Hg, Mg, Na	Ancient murals on the walls of a cave tested on-site in Dunhuang (Western China)	Mapping, depth profiling (normalized intensity vs. number of laser pulses)	X-ray diffraction (XRD)	Portable LIBS analyzers	[84]
42	Analysis and comparison of the penetration depth of different nanocomposites employed as biocides in the restoration of historical buildings and stone monuments	Ag, Ca, Mg, Ti	Limestone treated with an aqueous suspension of Ag and TiO_2_	Depth profiling (intensity vs. number of laser pulses)	-	Laboratory-made LIBS system	[87]

^1 ^The list shows the elements that were targeted and the elements whose signals were visible on the LIBS spectra. Molecular bands, discussed in the work or only visible on the LIBS spectra, are placed in parentheses. Explanation of abbreviations: LIA-QMS, laser-induced ablation-quadrupole mass spectrometry (LIA-QMS); GD-OES, glow discharge optical emission spectroscopy; EPMA, electron probe micro-analyzer; AES—Auger electron spectroscopy; SIMS—secondary ion mass spectrometry.

**Table 2:** List of applications of the LIBS technique in the studies of polymeric multilayer materials and corrosion studies of polymers employing commercial LIBS and laboratory-made LIBS systems.

No	Purpose of Studies	Analyte	Sample	Mode	Type of LIBS Device	Ref
1	Evaluating the potential of using LIBS for spatially resolved analysis and classification of laterally structured polymers, along with characterization of multilayer synthetic polymeric materials	C, H, O (C_2_ Swan)	3D-printed structured ABS/PLA sample; double-sided adhesive tape glued to S-wafer (PE liner, PAK adhesive layer, PVC support, and a second PAK)	Depth profiling (concentration vs. depth)	Commercial LIBS system	[30]
2	Examination of the oxidation of various PS samples subjected to controlled accelerated weathering conditions, especially for changes in PS that are not detectable using surface detection methods	C, Ca, Fe, H, K, N, Na, O, (C_2_), (CN)	Artificially aged polystyrene	Depth profiling (propagation of the oxidation into the samples; (intensity vs. number of laser pulses)	Laboratory-made LIBS system	[97]
3	Assessment of LIBS for on-site corrosion analysis of silicon rubber coatings used in high-voltage engineering applications	C, Fe, K Na, Si	Commercial silicone rubber samples	Depth profiling (intensity vs. number of laser pulses)	Laboratory-made LIBS system ^1^	[98]
4	Analysis of the relationship between the number of laser pulses and the topography of LIBS craters, as well as determining the correlation between the mass of ablated HDPE and the number of laser pulses	C, Na	Commercial HDPE samples	Depth profiling (intensity vs. depth)	Commercial LIBS system	[99]

^1^ the reference data were obtained using the EDS, XPS, and SIMS techniques. Explanation of abbreviations: acrylonitrile butadiene styrene (ABS), polylactic acid (PLA), polyethylene (PE), polyacrylate (PAK), and polyvinylchloride (PVC).
